# The complete mitochondrial genome of *Mactra quadrangularis* (Mactridae)

**DOI:** 10.1080/23802359.2021.1948365

**Published:** 2021-07-15

**Authors:** Chen Guan, Xin-Yu Zhao, Huan-Xin Zhang, Jun Chen, Tong-Fei Qu, Cheng-Zong Hou, Xue-Xi Tang, Ying Wang

**Affiliations:** a College of Marine Life Sciences, Ocean University of China, Qingdao, PR China; b Laboratory for Marine Ecology and Environmental Science, Qingdao National Laboratory for Marine Science and Technology, Qingdao, PR China; c College of Geography and Environment, Shandong Normal University, Jinan, PR China

**Keywords:** Bivalvia, Venerida, phylogenetic analysis

## Abstract

The whole mitochondrial genome sequence of *Mactra quadrangularis* (Reeve, 1854) was determined. It had a total length of 16,848 bp and it contained 12 protein coding genes, 2 ribosome RNA genes, and 22 transfer RNA genes. The base composition was 25.75% A, 20.82% G, 11.53% C, and 41.90% T, respectively. Furthermore, state codon of *ND4* was ATT; *ND1* and *CYTB* were ATA; *COX1* was GTG; *ND5*, *COX2*, *ND4L*, *ND6*, *ND2*, *COX3*, *ATP6*, and *ND3* were ATG. Phylogenetic analysis demonstrated that *M. quadrangularis* was most closely related to *Mactra chinensis.* The mitochondrial genome will provide reference for the further investigation and research of *M. quadrangularis*.

As an important edible seashore clam and seafood resource in the coastal areas of China, Japan, and South Korea, the surf clam *Mactra quadrangularis* (Reeve, 1854), sometimes also referred to as *Mactra veneriformis* (Reeve, 1854), belongs to Bivalvia, Venerida, Mactridae, *Mactra* (Linnaeus, 1767) (Hou et al. 2006; Luan et al. 2011; Nie et al. 2013; Zhu et al. 2019). We reconstructed the complete mitochondrial genome of *M. quadrangularis* based on Illumina paired-end sequencing data.

Samples were collected at Lianyungang, Jiangsu province, China (34.9497°N, 119.1886°E). The specimen is stored at Laboratory of Marine Ecology, Ocean University of China (specimen code OUCMLE09529; chenguan1021@163.com). Total genomic DNA was extracted from muscular tissue according to the CTAB method as detailed in (Mirimin and Roodt-Wilding 2015). We generated 400 bp paired-end reads from total genomic DNA by whole genome shotgun (WGS) sequencing using the Illumina NovaSeq platform (GenomeAnalyzer, Illumina, San Diego, CA). *De novo* assembly was conducted using A5-miseq version 20150522 (Coil et al. 2014) and SPAdes version 3.9.0 (Bankevich et al. 2012; Choi et al. 2020). The annotation was performed using GeSeq software (Tillich et al. 2017).

The complete mitochondrial genome of *M. quadrangularis* was submitted to GenBank and the GenBank accession number was MW691169. The raw data of *M. quadrangularis* were submitted to Sequence Read Archive (SAR) and the accession number was SRR14764610. The complete mitochondrial genome was 16,848 bp in size. The A + T base content (67.65%) was higher than the G + C content (32.35%). The base composition of the complete mitochondrial genome of *M. quadrangularis* was 25.75% for A, 20.82% for G, 11.53% for C, and 41.90% for T, respectively. The newly sequenced mitochondrial genome encodes for a total of 36 genes, including 12 protein-coding genes (PCGs), 2 rRNA genes, and 22 tRNA genes. In PCGs of *M. quadrangularis*, ATP8 gene is missing. Of the 12PCGs, *ND5*, *ND1*, *COX1*, *ND4L*, *COX3*, and *CYTB* had TAA as stop codons; while the other PCGs had TAG as stop codons. The state codon of *ND4* was ATT, that of *ND1* and *CYTB* was ATA, that of *COX1* was GTG, and that of *ND5*, *COX2*, *ND4L*, *ND6*, *ND2*, *COX3*, *ATP6*, and *ND3* was ATG.

To investigate the phylogenetic relationships of *M. quadrangularis*, four complete mitochondrial genomes of Mactridae (*Coelomactra antiquata* [Spengler, 1802], *Mactra chinensis* [Philippi, 1846], *Lutraria maxima* [Jonas, 1844], and *Lutraria rhynchaena* [Jonas, 1844]) and two complete mitochondrial genomes of Veneridae (*Meretrix* [Linnaeus, 1758]*, Meretrix lusoria* [Röding, 1798]), one mitochondrial genome of Arcticidae (*Arctica islandica* [Linnaeus, 1767]), as well as one mitochondrial genome of Cyrenidae (*Corbicula fluminea* [Müller, 1774]) were downloaded from GenBank and aligned using complete mitochondrial genome sequences (Figure 1). The maximum likelihood (ML) phylogeny was constructed based on the General Time Reversible + Invariant + gamma sites (GTR + I + G) model of nucleotide substitution with 1000 bootstrap replicates by Mega-X version 10.0.2 (Kumar et al. 2018). The ML tree analysis indicated that *M. quadrangularis* closely related to *M. chinensis* and *Coelomactra antiquata* (Figure 1). This published *M. quadrangularis* mitochondrial genome will provide evolutionary information in the Mactridae.

**Figure 1.**
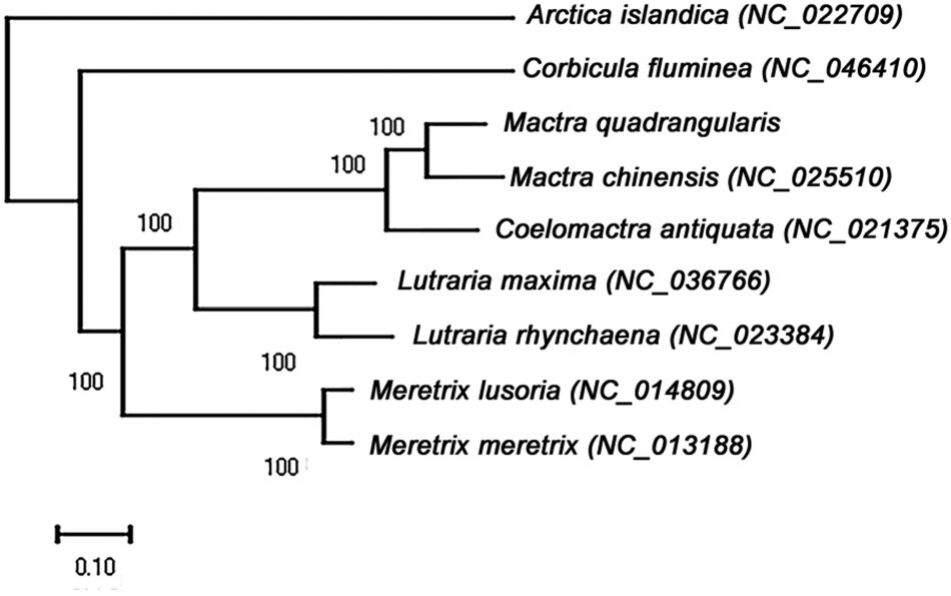
Maximum likelihood (ML) phylogenetic tree based on 9 complete mitochondrial genome sequences of Venerida. ML bootstrap values are shown above nodes. All the sequences were downloaded from NCBI GenBank.

## Data Availability

The data that support the findings of this study are openly available in GenBank of NCBI at https://www.ncbi.nlm.nih.gov/nuccore/MW691169/, reference number MW691169. BioProject accession number was PRJNA736184 at https://www.ncbi.nlm.nih.gov/bioproject/PRJNA736184. BioSample accession number at https://www.ncbi.nlm.nih.gov/biosample/SAMN19611295/ and Sequence Read Archive at https://www.ncbi.nlm.nih.gov/sra/SRR14764610.
